# Systemic Autoimmune, Rheumatic Diseases and Coinciding Psoriasis: Data from a Large Single-Centre Registry and Review of the Literature

**DOI:** 10.1155/2015/657907

**Published:** 2015-08-03

**Authors:** Anna Bazsó, Péter Szodoray, Ágnes Szappanos, Judit Korda, Patrícia Pálfi, Emese Kiss, Gyula Poór

**Affiliations:** ^1^National Institute of Rheumatology and Physiotherapy, Frankel Leó Utca 38-40, Budapest 1023, Hungary; ^2^Institute of Immunology, Rikshospitalet, Oslo University Hospital, 0027 Oslo, Norway

## Abstract

Psoriasis is a systemic immune-inflammatory disease characterized by chronic or recurrent skin symptoms, psoriatic arthritis, enthesopathy, and uveitis. Psoriasis has recently been published to appear with various autoimmune disorders, but the coexistence has been systematically reviewed by only few studies until now. In the present study, charts and electronic database of 4344 patients with various systemic autoimmune disorders, under regular medical control at our department, were reviewed retrospectively searching for association with psoriasis. Hereby, we demonstrate 25 psoriatic patients coinciding with various systemic autoimmune diseases. The coexistence of psoriasis and autoimmune diseases resulted in the worsening of the clinical outcome of the autoimmune diseases as indicated by higher frequency and dosages of glucocorticoid use, need for biologicals, and other comorbidities. These results suggest common environmental and genetic background as well as therapeutic possibilities in the future.

## 1. Introduction

Autoimmunity is characterized by the breakdown of self-tolerance leading to a state of abnormal humoral and cell-mediated responses agaissnst self-components. Psoriasis is an immune-inflammatory skin disease affecting 2-3% of the general population which can be associated with psoriatic arthritis (PsA), enthesopathy, uveitis, and an increased prevalence of cardiovascular morbidity [1]. The association between psoriasis and systemic autoimmune, rheumatic diseases is rare and little is known about its exact incidence. The pathogenesis of both disease entities involves genetic background and environmental triggers. A potential role of molecular mimicry has previously been described in the pathogenesis not only of autoimmune disease but also of psoriasis [2]. Several autoantigens have been implicated in psoriasis, amongst which are keratin 13 (K13), heterogeneous nuclear ribonucleoprotein-A1 (hnRNP-A1), and Rab coupling protein isoform 3 (FLJ00294) (RAB11FIP1), although the epidermal autoantigens have not been conclusively identified [3]. Underlying the importance of genetic associations, previously a clear correlation has been shown between psoriasis and risk of the development of diseases with autoimmune background, such as rheumatoid arthritis (RA), type 1 diabetes, celiac disease, or Crohn's disease, based on the single nucleotide polymorphism (SNP) analysis of the TNFAIP3 gene [4].

In this work, we demonstrate 25 patients with psoriasis and various systemic autoimmune diseases. Among the patients with autoimmune diseases included in our database we selected those who were associated with psoriasis. Our survey aimed to determine the prevalence of coinciding psoriasis in autoimmune conditions and whether psoriasis has an impact on the outcome of associated autoimmune diseases.

## 2. Materials and Methods

In this retrospective study medical charts and electronic database of patients, regularly followed at the National Institute of Rheumatology and Physiotherapy, were systematically reviewed searching for psoriasis as comorbidity. As psoriasis associated with the highest frequency to RA and SLE the same number of patients with and without psoriasis was selected and matched according to gender and age at onset, and as such case-control study could be performed. Patients in these subgroups were compared regarding the onset of the autoimmune diseases, clinical symptoms, and disease duration, as well as dose of corticosteroid and response to conventional and biological immunosuppressive therapies. In case of other autoimmune diseases only few patients belonged to subgroups with psoriasis; therefore a case-control study would not have been informative by statistical respect. Patients with psoriatic arthritis fulfilled the diagnostic criteria by laboratory markers, symptoms, and radiographic images and were distinguished from the joint manifestations of the coexisting autoimmune diseases.

### 2.1. Study Population

Out of the 4344 investigated patients (1450 with RA, 835 with Sjögren's syndrome, 807 with SLE, 486 with Raynaud's syndrome, 113 with undifferentiated connective diseases (UCTD), 313 with primary antiphospholipid syndrome (PAPS), 144 with polymyositis (PM), 127 with primary systemic vasculitis, 85 with systemic sclerosis, and 69 with mixed connective tissue diseases (MCTD)), 25 had coinciding psoriasis. Psoriatic arthritis was present in 14 cases. All patients fulfilled the corresponding classification criteria of the above-mentioned autoimmune diseases [1, 5–16]. Psoriasis coexisted with SLE (*n* = 8), rheumatoid arthritis (*n* = 5), primary Sjögren's syndrome (*n* = 5), primary Raynaud's syndrome (*n* = 4), primary systemic vasculitis (*n* = 3), APS (*n* = 2), systemic sclerosis (*n* = 2), UCTD (*n* = 1), polymyositis (*n* = 1), and MCTD (*n* = 1). Various other comorbidities also associate with different autoimmune diseases, such as hypertension, crystal arthritis, interstitial lung disease, ischemic heart disease, cataract, and glaucoma.

### 2.2. Data Collection

The clinical and laboratory data were collected from the institute's electronic patient databases from inpatient and outpatient visits. The following diseases were investigated: SLE, primary systemic vasculitis, PAPS, UCTD, primary Raynaud's syndrome, PM, systemic sclerosis, MCTD, primary Sjögren's disease, and RA. Each specific disease was treated as an outcome variable. All diagnoses for these conditions were recorded from September 2007 to November 2013. In our database the following data were detected: age at the onset of the autoimmune diseases, clinical symptoms, immune serology, associated diseases, disease duration, coexistence of psoriatic arthritis, actual clinical state, and average dose of corticosteroid, immune suppressive therapy, and response to the therapy.

### 2.3. Statistical Analysis

All statistical analyses were performed using IBM SPSS 20 software. Fisher's exact test was utilized to assess the average age of appearance of psoriasis and psoriatic arthritis and Mann-Whitney *U* test was performed to measure the average of corticosteroid usage.

## 3. Results

We determined the frequency of psoriasis in various autoimmune diseases and also assessed the rate of the psoriatic arthritis. We also aimed to compare demographic and disease-specific characteristics of RA and SLE with and without associating psoriasis.

There were 25 eligible individuals who fulfilled the criteria for psoriasis in the study population. The frequency of coinciding psoriasis was 0.99% in RA, 0.34% in SLE, 0.59% in Sjögren's syndrome, 0.82% in Raynaud's syndrome, 3.29% in systemic vasculitis, 6.3% in PAPS, 0.69% in PM, 2.35% in systemic sclerosis, 1.17% in UCTD, and 1.44% in MCTD. Out of the psoriatic cases 62.5% (*n* = 15) had psoriatic arthritis. Compared to the estimated vary of the population from 0.3% to 1% [15]. Psoriatic arthritis was diagnosed and distinguished from the musculoskeletal manifestations of the autoimmune diseases by the CASPAR criteria [16]. The median (min-max) age at autoimmune disease onset was 48 (24–68) years. Of those with psoriasis 12% were male and 88% were female. In 18 patients psoriasis developed first. In psoriatic patients who also suffered from different kinds of autoimmune diseases an increased rate of comorbidities was observed.

The second goal was to analyze demographic characteristics and the outcome of clinical symptoms in RA and SLE. In the case-control study the same number of patients with and without psoriasis was selected and matched out of our entire RA and SLE population. Demographic and disease-specific and treatment-associated data were compared in psoriatic and nonpsoriatic SLE and RA groups. The average age of appearance of psoriasis was 48 (24–68) years. The female to male ratio was 3 : 22 (12% and 88%). The appearance of psoriasis before and after the age of 40 years was similar (13/11 or 54% and 46%); however the frequency of psoriatic arthritis was significantly higher after 40 years of age (1/14 or 7% and 93%). Significantly higher doses of glucocorticoid (GC) were required in the SLE with psoriasis group (16.88 (10–30)) compared to SLE without psoriasis (11.4 (7.5–20)) (Tables 3 and 4). On the contrary in RA patients with psoriasis both the number of patients on GC and both the used GC doses were lower as compared to those with RA patients without associating psoriasis (Tables 1 and 2). The fact can be explained by differences in the usage of biological therapies, as all patients from the RA + psoriasis group were on TNF-alpha inhibitors, while in the control group only 1 patient received biological therapy. Both the SLE- and RA-associated psoriasis groups required intensified immune suppressive therapy. The association of psoriasis in both RA and SLE groups was characterized by worse laboratory markers, diseases outcome, and response to therapy. In the RA + psoriasis group 4 patients (80%) had other coinciding diseases, such as hypertension, neurofibromatosis, Sjögren's syndrome, and systemic sclerosis, as compared to the RA group where 2 patients (40%) had hypertension and Sjögren's syndrome. There were no significant differences of the immunological serology markers between the 2 groups. The responses to disease-modifying antirheumatic drugs (DMARDs) therapy were significantly worse in the RA + psoriasis group, since none of the patients responded or had side effects of the DMARDs. All patients of the RA + psoriasis group were treated with biologicals and 2 patients (40%) needed to switch to another biological therapy, while in the RA group only 1 patient (20%) received biological therapy and all (100%) patients were in remission (defined as Das28 <2.1) (Tables 1 and 2). In the SLE + psoriasis group 7 patients had comorbidities (87.5%) as compared to the 6 patients (75%) in the SLE-only group. Similar to the RA groups there were no significant differences in immunological markers between the 2 groups. Otherwise, in the SLE + psoriasis group 5 patients (62.5%) had relapse, 2 patients (25%) had worsening outcome, and only 1 patient (12.5%) was in remission when compared to the SLE group, where all patients were in remission. In the SLE + psoriasis group all patients received more than 1 immunosuppressive agent and the systemic lupus international collaborating clinics (SLICC) index was elevated in 7 patients (87.5%) compared to the SLE group (37.5%). The average dose of corticosteroids (PED) was 17.5 mg in the SLE + psoriasis group and 11.4 mg in the SLE-only group (Tables 3 and 4).

## 4. Discussion

The overlap between psoriasis and autoimmune diseases is unusual. Differential diagnostic problems may also occur. The prevalence of psoriasis in African Americans is 1.3% compared to the 2.5% in Caucasians [17]. In our database we found 25 patients (0.545%) with psoriasis from the 4344 autoimmune disorders. In the general population the prevalence of skin psoriasis is around 2-3%. The lower prevalence, found in our data base, can probably be attributed to concurrent existence of national tertiary dermatology centre taking care of psoriatic patients. Therefore, our centre followed those patients who suffer from psoriatic arthritis or other autoimmune disorders. We have confirmed that the overlapping psoriasis and autoimmune diseases result in the worsening of the autoimmune diseases, reflected by the increased corticosteroid usage, worse response to the therapy, and the appearance of other comorbidities. We have not observed a significantly higher tendency to develop more autoimmune diseases in patients with psoriatic arthritis. Particularly, in case of patients with RA and psoriasis, physicians should be aware of the possible development of psoriatic arthritis. Despite the unusual association there are several similarities in the innate and adaptive pathways and genetic and environmental—including the infections—background between psoriasis and autoimmune diseases.

Th1, Th17, and IL-22-producing CD4 T cells in psoriasis and autoimmunity have recently been supposed. The concept of molecular mimicry is based on a structural similarity between a pathogen and a metabolite and self-structures. Epidemiological findings show a strong correlation between EBV infection and the risk of developing sclerosis multiplex, SLE, RA, and primer SS [18]. There are some gene variants evaluated to be involved and are common in the pathogenesis of the four diseases of cytokine pathways (e.g., IRF5, STAT4, and TNFSF4) leading to the development of autoimmunity [19]. Regarding the genetic association between psoriasis and autoimmunity, TNF-alpha-induced protein 3 (TNFAIP3) has been shown to be a major candidate. Multiple variants of TNFAIP3 could modulate development of autoimmunity in different diseases. The TNFAIP3 gene region has been implicated in the susceptibility to multiple autoimmune diseases in Europe. SNPs ~ 185 kb upstream from the TNFAIP3 gene have a strong association with risk of RA, type 1 diabetes (T1D), celiac disease (CeD), and Crohn's disease [20]. Two independent studies regard the association between TNFAIP3 and SLE in a European cohort [21, 22]. Wu et al. have confirmed in a well-powered, genetic case-control study that both psoriasis and autoimmune diseases have complex genetic basis; multiple genes contribute to disease risk [23]. Overlapping of some gene locations of different autoimmune diseases has been known and supports common pathogenic gene variants (PTPN 22, Csk, PAG, PSTPIP1, PDCD1, SLC9A3R1, CARD15, and SUMO4) transcript within these diseases [24]. TNF-alpha polymorphism or TNF-alpha can increase the development of psoriasis or psoriatic arthritis. TNF inhibitors are effective in the treatment of psoriasis as well as in RA; however they can induce antinuclear antibodies and even lupus [25].

Interleukin-17 is a Th17 cytokine associated with inflammation, autoimmunity, and defence against some bacteria; it has been implicated in many chronic autoimmune diseases including psoriasis, Crohn's disease, autoimmune uveitis, SLE, ankylosing spondylitis, asthma, multiple sclerosis, and systemic sclerosis [26]. The pathophysiologic relevance of the IL-23-IL-17 axis in autoinflammatory diseases is highlighted by the clinical efficacy of antibodies targeting IL-23/IL-12 p40 and IL-17 in treating psoriasis, as well as the other systemic autoimmune diseases [27]. The level of IL-17 was significantly higher in serum of SLE patients than in normal controls, indicating that IL-17 may trigger the inflammatory process, although no correlation was found between serum IL-17 levels and disease manifestation or SLEDAI [28]. In general, the common IL-23-IL-17 axis may also initiate and maintain the coexistence of psoriasis and other systemic autoimmune diseases [27, 28].

The other prominent cytokine highlighting the common pathway is interferon- (IFN-) alpha, mainly produced by plasmacytoid dendritic cells (PDCs) [29]. IFN-alpha plays a pivotal role in the development of SLE, insulin-dependent diabetes mellitus (IDDM), or RA. In psoriatic lesions plasmacytoid dendritic cell infiltrations have been shown, indicating that IFN-alpha may contribute to the pathogenesis of these diseases [29–32].

The humoral and cellular immunity have been shown to act against endothelial antigens and moreover it has a greater risk of atherosclerosis in both diseases as RA and SLE. So, these processes highlighted the significance of autoimmunity in atherosclerotic processes. Both angiogenic and oxidative pathways have a common role in the pathophysiology of psoriasis and atherosclerosis. Psoriasis and autoimmune diseases have a strong relationship with lipid metabolism and oxidative stress. Heat shock protein (Hsp) and human Hsp are known as possible pathogenic links between infection and atherosclerosis, as well as infection and autoimmunity [33, 34]. However, the immune-mediated inflammatory disease (IMID) is a group of diseases without exact etiology, but involving common inflammatory pathways resulting in many diseases as psoriasis, psoriatic arthritis and atherosclerosis, also. Psoriasis and autoimmune diseases also associate with an increased risk of atherosclerosis. Activated inflammatory cells and proinflammatory cytokines contribute to the psoriatic lesions and the rupture of atherosclerotic plaque. Macrophages also interact with T cells and other cells via activation of the CD40-CD40 ligand pathway, which contributes to the atheromatous plaque rupture [34]. Anti-CD40 therapy has been shown to be efficacious in some autoimmune diseases, such as SLE, vasculitis, and pSS [35]. Several studies have shown the endothelial cell dysfunction, the deficiency of nitric oxide (NO)2, elevated endothelin 1 (ET-1), angiotensin II (Ang II), plasminogen activator inhibitor 1 (PAI-1), and cellular adhesion molecules. Furthermore, other common pathognomonic factors such as the Toll-like receptors (TLR2 and TLR4) play key roles in atherosclerosis. TLR2 and TLR4 bind to components of gram positive and gram negative bacteria which could be a pathognomonic factor in autoimmunity, as mentioned above [34].

Serum leptin, resistin, and lipocalin are increased in psoriasis patients and have a potential important role for developing insulin resistance and cardiovascular disease in psoriasis. An adipose tissue secreted cytokine, called adiponectin, is able to improve insulin resistance. Its serum level is decreased in psoriatic patients. The decreased level of different adipokines and Th17 cytokine has also associated in patients with psoriasis and autoimmune diseases, as well. However, leptin, adiponectin, resistin, and visfatin play a significant role in physiopathology of several inflammatory diseases. Moreover, all are involved strongly in other relevant inflammatory conditions and autoimmune disorders [36].

Nutritional compounds and drugs also trigger autoimmune disease. There are some data about the association of the early exposure to dietary cow's milk proteins and a decreased risk of T1D; tienilic acid, dihydralazine, and halothane have been reported to induce autoimmune hepatitis. Stress and smoking are also associated susceptibilities to many autoimmune diseases [37].

Moreover, there is also strong association between autoimmunity and psoriasis when analyzing new bone formations and bone erosion as cellular biomarkers such as osteoclast precursors; osteoprotegerin (OPG), matrix metalloproteinase 3, serum IL-6, and IL-2R alpha were found elevated. OPG is produced not only in bone but also in several other tissues, including the cardiovascular system, lungs, kidneys, immune tissues, and blood vessels. In vascular system, increased OPG levels may be related to endothelial lesion, intimal hyperplasia, smooth cell hypertrophy, or advanced plaque calcification [38].

## 5. Summary

We believe that further prospective, cohort studies are required to determine real frequency of psoriasis in various autoimmune diseases as well as the incidence of autoimmune diseases within psoriatic patients. Positive and negative prognostic factors are still to be identified. The characteristics and outcome of autoimmune diseases and psoriasis also have to be followed.

Our present findings suggest that psoriasis exists as a negative predictive factor for the clinical outcome of autoimmune diseases. Despite there being several similarities between the pathogenesis of psoriasis and autoimmune diseases it was surprising to find the low frequencies of coexistence.

Patients with RA and psoriasis are more likely to receive biological therapy, while patients with SLE and psoriasis need significant higher doses of glucocorticoids. TNF-*α* inhibitors are effective in the treatment of psoriasis; however they can induce antinuclear antibodies and even lupus. Therapeutic considerations have to be done in overlapping cases. Biologicals with different ways of action, for example, targeting of IL-17 and IL-23, dendritic cell suppression, might reduce activity of both diseases. Röhn et al. and de Carvalho et al. explored the fact that neutralization of IL-17 by passive and active vaccination may be a novel therapeutic approach for the treatment of SLE and atherosclerosis [39, 40]. Targeting IFN-alpha, RANK-RANKL, and CD40-CD40L system could also be beneficial for the prevention and early therapeutic intervention in psoriasis and other related autoimmune diseases [30, 34, 35, 38].

## Figures and Tables

**Table 1:** Patient characteristics with rheumatoid arthritis associated with psoriasis.

The age of RA onset	Comorbidity	Immunoserology	Actual clinical status	DMARD therapy	Response to DMARD	Corticosteroids (mg PED)	Biological therapy	Response to biology therapy	Psoriatic arthritis
63 yrs	Hypertension	ANA, ACPA	Remission	Sulfasalazine-leukopenia, oral methotrexate-gastrointestinal side effect, cyclosporine-with golimumab	Nonresponder	—	Golimumab	Remission	Axial

46 yrs	—	RF, ACPA, ANA	Remission	Oral methotrexate-ineffective, sulfasalazine-gastrointestinal side effect	Nonresponder	—	Etanercept	Remission	Peripheral

59 yrs	Systemic sclerosis	RF, ACPA, ANA	Remission	Chloroquine-gastrointestinal side effect sulfasalazine	Nonresponder	—	Adalimumab-ineffective, rituximab	Remission	Peripheral

32 yrs	Hypertension	RF, ACPA, ANA	Active polyarthritis	Oral methotrexate-bone marrow toxicity azathioprine-ineffective cyclosporine-ineffective combination of chloroquine, sulfasalazine, oral methotrexate-GI side effect subcutaneous methotrexate	Ineffective	>7.5 continuously	Adalimumab-ineffective etanercept-ineffective golimumab-ineffective	Nonresponder	Peripheral and axial

40 yrs	Sjögren's syndrome, neurofibromatosis	RF, ACPA, ANA, aCL IgM	Severe glandular symptoms, active polyarthritis	Leflunomide-ineffective, oral methotrexate-hepatotoxicity	Nonresponder	—	Etanercept	Just started	Peripheral

RA: rheumatoid arthritis, ANA: anti-nuclear antibody, ACPA: anti-citrullinated peptide antibody, aCL IgM: anti-cardiolipin antibody immunoglobulin M, DMARD: disease-modifying antirheumatic drugs, and PED: prednisolone equivalent dose.

**Table 2:** Patient characteristics with rheumatoid arthritis only.

At the age of RA onset	Comorbidity	Immunoserology	DMARD therapy	Response to DMARD	Corticosteroids (mg PED)	Biological therapy	Response to biology therapy
63 yrs	Hypertension	RF, ACPA	Sulfasalazine-ineffective, leflunomide-allergic side effect, oral methotrexate	Partial response	<7.5	Tocilizumab	Remission

46 yrs	—	RF, ACPA, ANA	Combination of oral methotrexate and chloroquine	Remission	10	—	—

59 yrs	Sjögren's syndrome	RF, ACPA, ANA	Combination of oral methotrexate and chloroquine	Remission	10	—	—

32 yrs	—	RF, ACPA, ANA	Combination of subcutaneous methotrexate and chloroquine	Remission	—	—	—

40 yrs	—	RF, ACPA	Subcutaneous methotrexate	Remission	—	—	—

RA: rheumatoid arthritis, ANA: anti-nuclear antibody, ACPA: anti-citrullinated peptide antibody, DMARD: disease-modifying antirheumatic drugs, and PED: prednisolone equivalent dose.

**Table 3:** Patient characteristics with SLE associated with psoriasis.

The age of SLE onset	Comorbidity	Immunoserology	Actual clinical status	Therapy	Arthritis psoriatica	Corticosteroids (mg PED)	SLICC
27 yrs	Lupus nephritis, autoimmune thyroiditis	ANA, aSSA, aSSB, aTPO	Relapsing, remission by MMF	Cyclophosphamide, mycophenolate mofetil, methylprednisolone	Peripheral	25	1

54 yrs	Hypertension, gout, ischaemic heart disease, COPD, cataract	ANA, a-dsDNA	Relapsing	Chloroquine, azathioprine, methylprednisolone, etanercept	Axial	12.5	4

45 yrs	—	ANA, a-SM, a-SSA	Remission	Oral methotrexate, leflunomide, chloroquine	—	—	0

24 yrs	Raynaud's syndrome, cutaneous vasculitis	ANA, a-dsDNA, a-SSA, aSSB, *β*2GPI IgG, hypocomplementaemia	Relapsing	Methylprednisolone, azathioprine, intravenous cyclophosphamide, IVIG, plasmapheresis,	—	30	1

33 yrs	Autoimmune thyroiditis, nephrotic syndrome	ANA, a-TPO, lupus anticoagulant, aCL IgM	Relapsing	Methylprednisolone, chloroquine, oral methotrexate,	—	10	1

57 yrs	Sjögren's syndrome	ANA, a-SSA, a-SSB, a-dsDNA, hypocomplementaemia	Relapsing	Oral methotrexate, methylprednisolone, sulfasalazine, intravenous cyclophosphamide	—	20	1

58 yrs	Sjögren's syndrome, urticaria vasculitis, ILD	ANA, a-dsDNA, a-chromatin, hypocomplementaemia	Worsening of ILD, psoriasis, immunoserology	Oral methotrexate, sulfasalazine, intravenous cyclophosphamide	Axial	30	1

29 yrs	Sjögren's syndrome	ANA, a-dsDNA, a-SSA, a-SSB, anti-RNP, a-U1RNP, a-TG	Worsening of psychosis, thrombocytopenia	Methylprednisolone	—	12.5	1

SLE: systemic lupus erythematosus, COPD: chronic obstructive pulmonary disease, ILD: interstitial lung disease, ANA: anti-nuclear antibody, ACPA: anti-citrullinated peptide antibody, a-SSA: anti-Sjögren's syndrome A antibody, a-SSB: anti-Sjögren's syndrome B antibody, anti-RNP: anti-ribonucleoprotein antibody, a-dsDNA: anti-double stranded deoxyribonucleic acid antibody, *β*2GPI IgG: beta 2 glycoprotein I IgG, aCL IgM: anti-cardiolipin antibody immunoglobulin M, aU1RNP: anti-U1 ribonucleoprotein antibody, a-TG: anti-thyroglobulin antibody, a-TPO: anti-thyreoid peroxidase antibody, MMF: mycophenolate mofetil, PED: prednisolone equivalent dose, and SLICC: systemic lupus international collaborating clinics.

**Table 4:** Patient characteristics with SLE only.

The age of SLE onset	Comorbidity	Immunoserology	Actual clinical status	Therapy	Corticosteroids (mg PED)	SLICC
27 yrs	—	ANA, a-SSA, a-SSB, a-U1RNP, hypocomplementaemia	Remission	Azathioprine, low-dose corticosteroid	13.75	0

54 yrs	Gout, diabetes mellitus, COPD, ischemic heart disease	ANA, a-dsDNA, a-chromatin	Remission	Low-dose corticosteroid	12.5	1

45 yrs	—	ANA, a -Sm, a-SSA, a-U1RNP, aCL IgM	Remission	—	20	2

24 yrs	Raynaud's syndrome, Sjögren's syndrome, Hashimoto-thyroiditis, seronegative rheumatoid arthritis (erosive polyarthritis), idiopathic thrombocytopenic purpura	ANA, a-ENA, a-SSA, a-dsDNA, a-Sm, a-TPO	Remission	—	10	0

33 yrs	Sjögren's syndrome	ANA, a-dsDNA, a-SSA, a-SSB, aCL IgM	Remission	—	8.7	0

58 yrs	Sjögren's syndrome,	ANA, a-dsDNA, a-SSA, a-SSB,	Remission	—	8.75	0
	type 2 diabetes mellitus					

57 yrs	Rheumatoid arthritis, sacroiliitis	ANA, a-ACPA, a-dsDNA, a-Sm, a-U1RNP	Remission	—	7.5	0

29 yrs	Rheumatoid arthritis, asthma bronchial, aseptic femur-head necrosis, cataract, glaucoma	ANA, a-ACPA, a-dsDNA	Remission	—	10	2

SLE: systemic lupus erythematosus, COPD: chronic obstructive pulmonary disease, ILD: interstitial lung disease, ANA: anti-nuclear antibody, a-SSA: anti-Sjögren's syndrome A antibody, a-SSB: anti-Sjögren's syndrome B antibody, a-chromatin: anti-chromatin antibody, anti-RNP: anti-ribonucleoprotein antibody, a-dsDNA: anti-double stranded deoxyribonucleic acid antibody, anti-Sm: anti-Smith antibody, aCL IgM: anti-cardiolipin antibody immunoglobulin M, a-U1RNP: anti-U1 ribonucleoprotein antibody, a-TPO: anti-thyreoid peroxidase antibody, MMF: mycophenolate mofetil, PED: prednisolone equivalent dose, and SLICC: systemic lupus international collaborating clinics.
